# Relationship Between Vesicoureteric Reflux and Nephrocalcinosis in Children: A Case-Control Study at a Tertiary Medical Center in Saudi Arabia

**DOI:** 10.7759/cureus.30650

**Published:** 2022-10-25

**Authors:** Ahmed S Al-Zahrani, Jaffer S Al-Faraj, Maria Blesilda B Llaguno, Ali M Alhussain

**Affiliations:** 1 Pediatric Radiology, King Abdullah Specialist Children's Hospital, Riyadh, SAU; 2 Nursing, College of Applied Medical Sciences, King Faisal University, Hofuf, SAU; 3 Clinical Research, Maternity and Children Hospital–Al-Ahsa, Hofuf, SAU

**Keywords:** nephrocalcinosis, vesicoureteric reflux, pediatric congenital urogenital abnormality, pediatric neuroradiology, pediatric radiology

## Abstract

Background:* *Vesicoureteral reflux (VUR), one of the most common pediatric congenital urogenital abnormalities, refers to the abnormal backflow of urine from the urinary bladder back into the ureter or to the kidney. This causes urinary tract infections. Nephrocalcinosis (NC) refers to abnormal deposits of calcium within the renal parenchyma and/or in the renal cortex. Patients with NC are mostly asymptomatic and severe disease may progress to renal failure. Early diagnosis through examinations such as radiography, computed tomography, and ultrasonography, is crucial for therapeutic treatment. Ultrasonography is the preferred method for scanning and grading nephrocalcinosis in children, primarily because it emits no radiation. This study aimed to increase the body of knowledge regarding VUR and nephrocalcinosis by determining its prevalence and assessing the relationship between VUR and nephrocalcinosis in children presenting at our institution.

​​​​Methods:A case-control study was conducted using data from the medical records of 632 children younger than 14 years in a tertiary medical center in Riyadh, Saudi Arabia. Eligible participants were assigned to two groups: Group 1 consisted of 316 patients with VUR, while Group 2 consisted of 316 sex- and age-matched patients without VUR. The difference in the prevalence of nephrocalcinosis between the two groups was assessed. Frequency and percentage were used to present the categorical variables; Pearson product-moment correlation was utilized to establish the association between VUR and nephrocalcinosis. Statistical significance was established at p<0.05.

​​​​​Result: Only two cases in Group 1 were positive for nephrocalcinosis (0.63%, one male and one female), while four cases in Group 2 were positive for the condition (1.26%, two males and two females). There was no significant difference in the incidence of nephrocalcinosis between the two groups (p=0.873), indicating no relationship between VUR and nephrocalcinosis in children.

Conclusion: There is no relationship between VUR and nephrocalcinosis in children under the age of 14 years.

## Introduction

Vesicoureteral reflux (VUR) refers to the abnormal backflow of urine from the urinary bladder back into the ureter or to the kidney, either unilaterally or bilaterally. It accounts for about 25% to 40% of urinary tract infections (UTIs) in children, with no significant difference in sex prevalence among patients with UTIs, except in infancy [1]. The VUR is one of the most common congenital urogenital abnormalities in children and is diagnosed through a voiding cystourethrogram (VCUG), usually after the patient has had a UTI.

The VCUG is graded from 1 (mild) to 5 (severe). Recurrent UTIs and reflux nephropathy, which lead to renal scarring, are the two most common complications of VUR. Renal scarring can, however, occur with UTIs in the absence of VUR or with VUR in the absence of a UTI [2]. Vesicoureteral reflux has been reported to have the lowest prevalence among Black children and noted in approximately 10% to 51.4% of children being investigated for a UTI for the first time [3].

Nephrocalcinosis is defined as abnormal deposits of calcium within the renal parenchyma and/or in the renal cortex [4]. The condition is divided into two types, based on where the calcium deposits are located in the body: medullary nephrocalcinosis affects the renal medulla (approximately 97% of patients) and cortical nephrocalcinosis affects the renal cortex [5]. The definite prevalence and incidence rates of nephrocalcinosis are difficult to calculate due to the high percentage (up to 40%) of incidentally discovered cases [4]. Furthermore, nephrocalcinosis was found to be high among neonates with a gestational age of less than 32 weeks or birthweight less than 1500 g [6]. The common etiologies for medullary nephrocalcinosis are hyperparathyroidism, medullary sponge kidney, renal tubular acidosis, and hypervitaminosis D. The common etiologies for cortical nephrocalcinosis are renal cortical necrosis, chronic glomerulonephritis, and chronic renal allograft rejection. Many other diseases have been described in association with nephrocalcinosis. In one study, autonomous hyperparathyroidism, distal renal tubular acidosis, and medullary sponge kidney were described as the most frequent clinical diagnoses in patients with radiological nephrocalcinosis [5].

Patients with nephrocalcinosis are mostly asymptomatic, and severe disease may progress to renal failure. Early diagnosis is crucial for therapeutic treatment and the primary aim of early treatment is to slow down or stabilize the course of the condition; cures are rare [7]. Nephrocalcinosis can progress to urolithiasis, with the development of a renal stone that may lead to abdominal pain with hematuria. This condition can be diagnosed by radiography, computed tomography, and ultrasonography. Of the three, ultrasonography is the most preferred and reliable method for scanning and grading nephrocalcinosis in children [6] because it has no radiation hazards, is portable, and is relatively inexpensive compared with computed tomography [7].

A search for English-language articles in the last 40 years using the keywords "vesicoureteral reflux" and "nephrocalcinosis," revealed that no study has been conducted that specifically explored the association between VUR and nephrocalcinosis. It has been reported that children with VUR are at high-risk to experience recurrent UTIs and the probable mechanism of nephrocalcinosis was the stagnation of urine in collecting tubules favoring the precipitation of calcium salts. Although the prognosis of nephrocalcinosis is not always bad, however, it can lead to renal dysfunction if not treated. Since there is a dearth of literature about the said conditions, this study aimed to increase the body of knowledge regarding VUR and nephrocalcinosis by determining the prevalence and assessing the relationship between VUR and nephrocalcinosis. 

## Materials and methods

Study area/setting

This study was conducted at the King Abdullah Specialist Children's Hospital (KASCH) at King Abdul-Aziz Medical City, Riyadh, Saudi Arabia. This institution is one of the largest specialized children’s hospitals in the Middle East with a 600-bed capacity and falls under the auspices of the Ministry of National Guard-Health Affairs in Riyadh, Saudi Arabia. 

Study participants

Patients younger than 14 years who underwent VCUG and renal or abdominal ultrasonography examination supervised by a pediatric radiology consultant or fellow in the pediatric radiology department at our institution, between January 2018 and May 2021, yielding either positive or negative VUR, were included in this study. Patients in the negative VUR group had the additional inclusion criteria of being sex- and age-matched to those in the positive VUR group.

The exclusion criteria were as follows: those aged 14 years and older, no VCUG, no renal or abdominal ultrasonography, or a known etiology for nephrocalcinosis.

 ​​​​​Study design

A case-control design was utilized for this study to determine if there is a relationship between VUR and nephrocalcinosis among children.

Sampling technique

Purposive sampling was utilized for this study. The study population consisted of 1518 patients younger than 14 years who were identified through the Picture Archiving and Communication System (PACS) and the BESTCare 2.0 Hospital Information System (BESTCare Inc., Torrance, CA, USA). A total of 632 cases were included in the study after applying the specified inclusion and exclusion criteria. Those who qualified for inclusion were assessed and assigned to one of two groups: Group 1 consisted of 316 patients with VUR, while Group 2 consisted of 316 sex- and age-matched patients without VUR. The remaining 886 from the total population were excluded from the study.

Data collection methods

Data collection commenced after obtaining approval from the Institutional Review Board (IRB) of King Abdullah International Medical Research Center (approval number: H-01-R-005). Then, a medical records analysis was performed utilizing the patients’ clinical data from medical charts and images seen from January 2018 and May 2021 based on the set inclusion criteria. The data were extracted into an Excel sheet (Microsoft Corp., Redmond, WA, USA) and coded using consecutive numbers instead of the patient's medical record numbers (MRNs) to ensure confidentiality. The sample was categorized into groups 1 or 2. All study data were collected through PACS and BESTCare 2.0 to achieve the main objectives. The patients’ medical charts were likewise utilized to exclude any positive nephrocalcinosis cases with a well-known etiology to avoid or minimize the potential for bias and confounding effects. 

Data management and analysis plan

Data were analyzed using the Statistical Package for Social Sciences (SPSS) version 21 (IBM Corp., Armonk, NY, USA). The categorical variables for the positive VUR group included age, sex, presence of left/right reflux, grading of the left/right kidney, and presence/absence of nephrocalcinosis. The categorical variables for the negative VUR group included age, sex, and presence/absence of nephrocalcinosis. The data were documented and presented as frequencies and percentages. The prevalence of nephrocalcinosis in the two groups was then assessed. To determine the association between VUR and nephrocalcinosis, the Pearson product-moment correlation was utilized. Statistical significance was established at p<0.05.

Ethical considerations 

To maintain patients’ privacy and confidentiality, all MRNs were replaced and coded with serial numbers. The research team had exclusive access to the medical charts.

## Results

The sample size of 632 (316 cases for each group) was selected based on the inclusion and exclusion criteria set by the researchers. The cases were extracted from among the 1518 VCUG cases documented at the pediatric radiology department at KASCH during the study period. Study findings are presented based on the patient profiles from both groups according to age (subdivided into six categories) and sex. The findings of those with positive VUR are presented based on the side (bilateral, isolated right-sided, or isolated left-sided) and severity of the reflux. Furthermore, findings for those without VUR are discussed.

General patient profile

Patient Profile According to Age

The selected cases from both groups were further subdivided into six categories as shown in Table 1.

**Table 1:** Frequency and percentage distribution of patients according to age

		Frequency	Percentage	Valid %	Cumulative Percentage
Valid	1 Day to <1 Week	46	14.6	14.6	14.6
	1 Week to <1 Month	57	18.0	18.0	32.6
	1 Month to <1 Year	102	32.3	32.3	64.9
	1 Year to <5 Years	60	19.0	19.0	83.9
	5 Years to <10 Years	35	11.1	11.1	94.9
	10 Years to <14 Years	16	5.1	5.1	100.0
	Total	316	100.0	100.0	

Patient Profile According to Sex

 As seen in Table 2, most patients were male (58.5%).

**Table 2:** Frequency and percentage distribution of patients according to sex (Group 1)

		Frequency	Percentage	Valid Percentage	Cumulative Percentage
Valid	Male	185	58.5	58.5	58.5
	Female	131	41.5	41.5	100.0
	Total	316	100.0	100.0	

Patient profile with VUR (Group 1)

Patient Profile Based on the Side of the Reflux

For those with VUR, there were 136 (43.04%) cases of bilateral reflux and 90 cases of isolated right-sided and isolated left-sided patients (28.48% for each) (Table 3).

**Table 3:** Patient profile based on the side of the reflux (Group 1)

Side of Reflux	Frequency	Percentage
Bilateral reflux	136	43.04
Isolated right-sided	40	24.48
Isolated left-sided	38	24.48
TOTAL	316	100

Patient Profile Based on the Severity of the Reflux

The cases were subdivided into six categories on each side (Table 4).

**Table 4:** Patient profile according to the severity of reflux (Group 1) * Considered positive because the other side has reflux in the same patient

Categories	RIGHT SIDE		LEFT SIDE	
	Frequency	Percentage	Frequency	Percentage
Category 1: Grade 0, no reflux appreciated*	90	28.5	90	28.5
Category 2: Grade 1 reflux	40	12.7	37	11.7
Category 3: Grade 2 reflux	38	12	43	13.6
Category 4: Grade 3 reflux	41	13	39	12.3
Category 5: Grade 4 reflux	48	15.2	40	12.7
Category 6: Grade 5 reflux	59	18.7	67	21.2
TOTAL	316	100	316	100

Patient Profile According to the Presence of Nephrocalcinosis

Table 5 shows that only 0.6% of the cases had nephrocalcinosis, while 99.4% did not have it.

**Table 5:** Frequency and percentage distribution of patients with nephrocalcinosis (Group 1)

		Frequency	Percentage	Valid Percentage	Cumulative Percentage
Valid	No	314	99.4	99.4	99.4
	Yes	2	.6	.6	100.0
	Total	316	100.0	100.0	

Patient Profile Based on the Side of the Reflux (Group 1: Right and Left Positive VUR) 

Table 6 shows that most patients with right-sided VUR (18.7%) had category 6 reflux. The same result is also seen for those with left-sided VUR, as shown in Table 7.

**Table 6:** Frequency and percentage distribution of patients with right VUR (Group 1) * Considered positive because the other side has reflux in the same patient VUR: Vesicoureteral reflux

		Frequency	Percentage	Valid Percentage
Category 1: Grade 0, no reflux appreciated*	90		28.5	28.5
Category 2: Grade 1 reflux	40		12.7	12.7
Category 3: Grade 2 reflux	38		12.0	12.0
Category 4: Grade 3 reflux	41		13.0	13.0
Category 5: Grade 4 reflux	48		15.2	15.2
Category 6: Grade 5 reflux	59		18.7	18.7
Total	316		100.0	100.0

**Table 7:** Frequency and percentage distribution of patients with left VUR (Group 1) * Considered positive because the other side has reflux in the same patient VUR: Vesicoureteral reflux

	Frequency	Percentage	Valid Percentage
Category 1: Grade 0, no reflux appreciated*	90	28.5	28.5
Category 2: Grade 1 reflux	40	12.7	12.7
Category 3: Grade 2 reflux	38	12.0	12.0
Category 4: Grade 3 reflux	41	13.0	13.0
Category 5: Grade 4 reflux	48	15.2	15.2
Category 6: Grade 5 reflux	59	18.7	18.7
Total	316	100.0	100.0

Patient Profile Based on the Side of the Reflux (Group 1)

**Figure 1:**
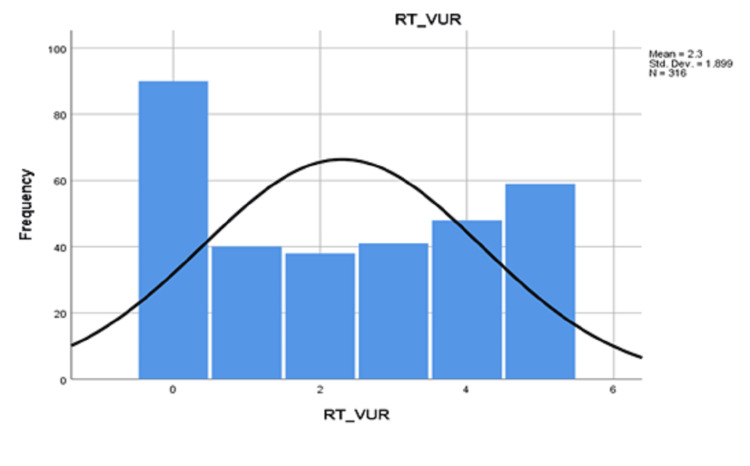
Category of right vesicoureteral reflux (RT_VUR) Std.Dev: Standard deviation

**Figure 2:**
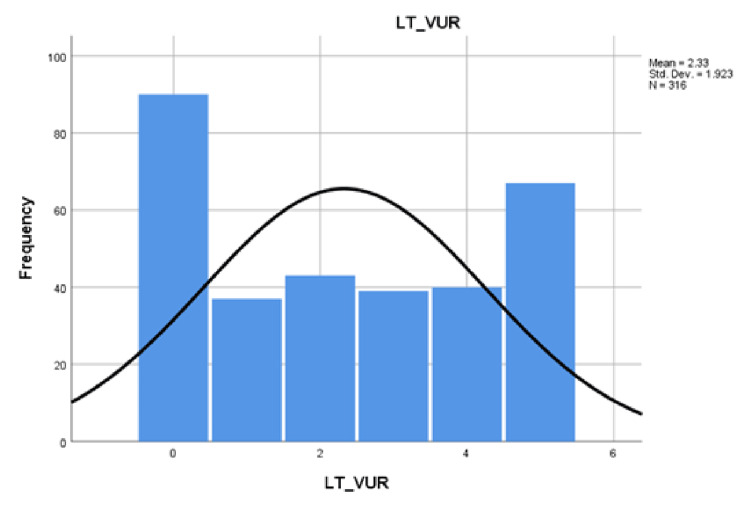
Category of left vesicoureteral reflux (LT_VUR) Std.Dev: Standard deviation

The mean reflux category for cases with right VUR was 2.3 ± 1.899 (Figure 1) and that of left VUR was 2.33 ± 1.923 (Figure 2).

Patient profile without VUR (Group 2)

Patient Profile According to the Presence of Nephrocalcinosis

Table 8 shows that only 0.6% of cases in Group 1 had nephrocalcinosis, while 99.4% did not. Table 9 likewise shows that for those without VUR, 1.3% of cases had nephrocalcinosis, while 98.7% did not.

**Table 8:** Frequency and percentage distribution of patients with nephrocalcinosis (Group 1)

		Frequency	Percentage	Valid Percentage	Cumulative Percentage
Valid	No	314	99.4	99.4	99.4
	Yes	2	.6	.6	100.0
	Total	316	100.0	100.0	

**Table 9:** Frequency and percentage distribution of patients with nephrocalcinosis (Group 2)

		Frequency	Percentage	Valid Percentage	Cumulative Percentage
Valid	No	312	98.7	98.7	98.7
	Yes	4	1.3	1.3	100.0
	Total	316	100.0	100.0	

Association between VUR and nephrocalcinosis

There was no significant difference in the incidence of nephrocalcinosis between the positive and negative VUR groups (p=0.873), indicating no relationship between VUR and nephrocalcinosis in children under the age of 14 years.

**Table 10:** Association between VUR and nephrocalcinosis VUR: Vesicoureteral reflux

		Negative Nephrocalcinosis	Positive Nephrocalcinosis
Negative Nephrocalcinosis	Pearson Correlation	1	-.009
	p-value		.873
	N	316	316
Positive Nephrocalcinosis	Pearson Correlation	-.009	1
	p-value	.873	
	N	316	316

## Discussion

This study noted that VUR was detected in more than one-fifth of children who had undergone a VCUG examination, in contrast to a study that reported the detection of VUR in more than one-third of young children presenting with a UTI [2]. The difference in incidence is considered to be due to the researchers of the current study including cases with other complaints, not only those with UTIs. It is noteworthy that VUR is predominant among infants. In fact, in a cohort of pediatric patients with UTI, including 68% of infants, VUR was diagnosed in 33% of cases [8]. The most common age group affected is usually between two to 12 months [9]. In contrast, another study suggested that the mean age of the patients was 36.5 months; 29% of them were below one year of age [10].

It is likewise worth noting that in our study cohort, males were more affected than females, with a male-to-female ratio of 1.5:1. Although similar findings have been reported [9,10], some studies have reported either no sex bias or female predominance [8,11]. In this study, VUR demonstrated bilaterality in 43% of patients, compared with 28% for both isolated right- and left-sided VUR, which is consistent with the findings of studies conducted in Nepal, Sudan, Saudi Arabia, and Brazil (60%, 56%, 61%, and 54%, respectively) [10-13]. Most cases had severe VUR of grades 4 and 5 (68%), followed by mild (grades 1 and 2, 50%) and moderate (grade 3, 25%) VUR. This finding is consistent with another study that showed a severe reflux rate of 64% [13], but it contrasts sharply with several other studies which showed a high incidence of low-grade VUR [10,11,14,15].

Nephrocalcinosis occurred in only 0.6% of the cases in the positive VUR group and 1.26% of those in the negative VUR group. It can be divided into three categories: molecular nephrocalcinosis, involving an increase in renal intracellular calcium without any crystal formation and essentially reflecting the renal dysfunction of hypercalcemia; microscopic nephrocalcinosis, in which calcium phosphate (CaP) or calcium oxalate (CaOX) crystals are visible on light microscopy, but not radiologically; and macroscopic nephrocalcinosis, when calcification is visible radiologically or on ultrasound scans [16,5]. As with the sites involved, it can either be classified as cortical or medullary [17]. Cortical nephrocalcinosis is rare and usually results from severe destructive disease of the cortex, such as in chronic glomerulonephritis, though often in association with another factor, such as increased calcium ingestion, acute cortical necrosis, chronic pyelonephritis, or trauma [18], autosomal recessive polycystic kidney disease, primary and secondary oxalosis, chronic renal allograft rejection, or benign nodular cortical nephrocalcinosis [5]. Medullary nephrocalcinosis, on the other hand, is the typical form seen in 98% of cases of human nephrocalcinosis. It forms clusters of calcification around each renal pyramid. It is common in patients with metabolic conditions (several of which are monogenic diseases) that predispose them to renal calcium stones. Knowing which genes are involved can help to shed light on the mechanisms behind nephrocalcinosis [17]. There was no significant difference in incidence between the two groups (p=0.873) in our study, indicating that there was no relationship between VUR and nephrocalcinosis in children under 14 years of age.

Our study is limited by the lack of other studies evaluating the relationship between VUR and nephrocalcinosis, preventing us from being able to make meaningful comparisons. Likewise, the small sample size and being a single-center study limited the study findings. We recommend that similar studies involving more patients be performed in other research centers or hospitals to further build upon the body of knowledge about the association between VUR and nephrocalcinosis.

## Conclusions

This study found that VUR is more predominant among infants younger than one year of age than among older children. More males are affected, with a male-to-female ratio of 1.5:1. Most patients had severe VUR. Only 0.6% of the patients in the positive VUR group had nephrocalcinosis, while the negative VUR group had a 1.26% prevalence of nephrocalcinosis. We, therefore, conclude that there is no relationship between VUR and nephrocalcinosis in children under the age of 14 years.
